# Evidence-aware comparison of sequence-centric machine learning for antibody discovery and optimization

**DOI:** 10.1093/bib/bbag459

**Published:** 2026-08-25

**Authors:** Jianxiong Zhao, Xiaoyun Yan, Junhai Han, Hao Xie

**Affiliations:** School of Life Science and Technology, Key Laboratory of Developmental Genes and Human Disease, Southeast University, 2# Dongda Road, Nanjing, Jiangsu, 210031, China; School of Life Science and Technology, Key Laboratory of Developmental Genes and Human Disease, Southeast University, 2# Dongda Road, Nanjing, Jiangsu, 210031, China; School of Life Science and Technology, Key Laboratory of Developmental Genes and Human Disease, Southeast University, 2# Dongda Road, Nanjing, Jiangsu, 210031, China; School of Life Science and Technology, Key Laboratory of Developmental Genes and Human Disease, Southeast University, 2# Dongda Road, Nanjing, Jiangsu, 210031, China

**Keywords:** antibody design, sequence-centric machine learning, antibody–antigen modeling, generative modeling, benchmarking and validation

## Abstract

Sequence-centric machine learning is increasingly used across antibody discovery and optimization, from repertoire-scale representation learning to target-aware scoring and generative design. Cross-study comparison remains difficult because methods differ in target conditioning, molecular output, dataset construction, benchmark design, and validation evidence. We therefore conducted a structured mapping of primary antibody machine-learning studies reported from 2020 to 30 June 2026 and organized the literature using a three-layer functional stack—foundation priors, scorer–rankers, and generator–optimizers—and three analytical axes: conditioning interface, output granularity, and validation evidence profile. A standardized method-level synthesis is complemented by representative anchor cases and four framework-guided audits showing how split units, recovery metrics, computational proxies, and sequence novelty can change the interpretation of headline results. The framework separates training supervision and internal evaluation from complementary domains of independent validation and links increasing molecular commitment to broader evaluation needs. We also provide an operational reporting checklist for auditing datasets, splits, negative construction, generative evaluation, experimental attrition, and resource availability. The framework is intended as a comparative audit scaffold for evidence-aware interpretation rather than as a universal performance ranking or formal benchmarking standard.

## Introduction

Therapeutic antibodies and *in vitro* diagnostic reagents are central to modern biomedicine. Classical discovery platforms, including hybridoma technology and phage display, remain indispensable because they provide experimentally grounded selection and validation [1, 2]. However, discovery is often followed by affinity maturation, specificity testing, and developability assessment, and liabilities such as poor expression, instability, aggregation, high viscosity, or polyspecificity may emerge only after substantial downstream characterization. These constraints motivate computational methods for earlier triage and optimization.

More broadly, machine learning is increasingly used to extract task-relevant information from biological sequences. Sequence-derived representations and learned features have been explored across proteomics and protein-property prediction, including interpretation of proteomic sequence information and prediction of experimentally relevant properties such as crystallization propensity [3, 4]. Antibody modeling is a particularly demanding instance of this broader sequence-centric setting because sequence variation must ultimately be interpreted in relation to molecular recognition, target context, and candidate-level utility.

Large antibody repertoires and protein language models now support reusable antibody representations, candidate ranking, and sequence generation before wet-lab testing, yet reported advances remain difficult to compare [5–10]. Studies expose models to different forms of target context, construct positives, and negatives under different assumptions, split data at different biological units and evaluate outputs that range from scalar scores to paired VH/VL sequences. The supporting evidence also varies across computational benchmarks, selection readouts, quantitative binding, specificity, experimentally resolved complexes, and developability assays. Consequently, the same metric name or broad task label can describe substantially different generalization problems and levels of molecular commitment.

In this review, sequence-centric denotes methods in which antibody amino-acid sequence remains a primary modeling object—as an input, scored or optimized candidate, or generated output—even when antigen structures, molecular surfaces, predicted complexes, or other modalities are introduced. We compare the literature through three questions: what biological or target context is available to the method, what molecular or decision object it commits to producing or ranking, and what evidence supports the claimed capability. These questions define the three axes developed in Section 2 including conditioning interface, output granularity, and validation evidence profile.

The review is an evidence-aware methodological comparison. We use structured literature mapping, standardized method-level extraction, and selected analytical anchor cases to examine when published results are directly comparable and when their interpretation must be narrowed. Structure-conditioned and multimodal methods are included as boundary cases when antibody sequence remains central to the scored, optimized, or generated object. The review is therefore intended to bridge methodological development and experimental interpretation, supporting readers who design antibody machine-learning models, evaluate model-assisted discovery workflows, or assess the comparability of reported performance, design claims, and validation evidence across studies.

### Review scope and study selection

We defined the core scope around sequence-centric machine-learning methods in which antibody sequence is a primary modeling object. Eligible studies included antibody representation learning and pretraining; antibody–antigen interaction, specificity, affinity-change, or developability scoring; humanization and sequence optimization; and generative design of Complementarity-Determining Regions (CDRs), variable regions, or paired heavy–light chains. Nanobody methods were included when they addressed the same sequence-to-target modeling problem. Structure-conditioned or multimodal methods were retained when they scored, optimized, or generated antibody sequences and provided informative boundary cases for the framework. Studies focused exclusively on structure prediction, docking, or generic protein modeling without a direct antibody discovery or engineering task were excluded from the core method map.

The literature mapping was updated through 30 June 2026 and focused on primary methodological studies reported from January 2020 onward. PubMed, arXiv, and bioRxiv were searched using combinations of antibody, immunoglobulin, nanobody, or B-cell receptor terms with machine learning, deep learning, language model, foundation model, transformer, generative model, diffusion, or reinforcement learning, together with discovery, design, optimization, humanization, binding, interaction, specificity, affinity, or developability. Earlier studies were retained when needed to define benchmark practices, evidence types, or foundational datasets. Peer-reviewed original studies were prioritized; technically substantive preprints were included when they introduced a distinct methodological capability or occupied a comparison space not yet represented by a peer-reviewed method, with publication status recorded explicitly.

Eligible methods were mapped before the detailed case studies were selected. Standardized extraction covered the primary task, model-stack layer, input and target-conditioning modalities, structural information, paired-chain modeling, molecular output, training and evaluation data, split or generalization unit, negative construction where applicable, benchmark and reported metrics, validation evidence profile, independent experimental validation type, failure or attrition reporting, developability assessment and code/model/data availability. Table 1 provides the broader landscape synthesis, Section 3 then examines selected analytical anchor cases chosen to span different stack layers and regions of the three-axis comparison space.

**Table 1:** Standardized method-level comparison of sequence-centric antibody machine-learning studies included in the structured literature mapping. The extracted fields summarize model role, input, and target conditioning, structural, and paired-chain modeling, molecular output, data, and benchmark construction, reported quantitative evaluation, validation evidence, attrition, developability, and resource availability.

Method/study	Year/status	Primary task	Stack layer	Input modality	Antigen-conditioned?	Structural information used?	Paired VH/VL modeled?	Output/design target	Training/evaluation data reported	Data scale reported	Split/generalization strategy reported	Negative construction	Benchmark type/reported metrics	Reported quantitative outcome(s)	Independent validation/assay type	Evidence profile	Failure/attrition reported?	Developability/candidate-viability reported?	Code/model/data availability	Major comparison limitation
BioPhi/Sapiens and OASis [11]	2022; peer reviewed	Antibody humanization and humanness evaluation	Generator–optimizer/scorer–ranker	Antibody VH/VL amino-acid sequences	No antigen conditioning	No explicit structure input for core Sapiens/OASis workflow	No; chain-level sequence editing/evaluation	Humanized antibody sequence; humanness score	OAS-derived human antibody repertoires; *in silico* humanization benchmark	Sapiens trained on OAS; benchmark of 177 antibodies reported	Sequence/humanization benchmark; not a target-generalization split	N/A	Humanness/humanization benchmark; comparison with expert-like redesigns	Humanness, sequence preservation and OASis-related humanness metrics reported	No independent antigen-binding validation as the core evidence	COMP	Not a wet-lab attrition study	Humanness/immunogenicity-risk proxy; not experimental immunogenicity	Open-source platform and web resource reported	Humanization/humanness does not establish antigen binding, specificity, or experimental candidate utility
AntiBERTa [6]	2022; peer reviewed	Antibody-specific self-supervised representation learning; paratope fine-tuning case study	Foundation prior	Antibody/BCR heavy- and light-chain sequences	No antigen conditioning in pretraining; paratope task uses antibody labels	Attention/contact analyses use structural interpretation; structure not a pretraining input	No; chain-level model inputs	Contextual antibody embeddings; paratope prediction after fine-tuning	OAS BCR sequences; SAbDab-derived paratope fine-tuning data	42 M unpaired heavy-chain and 15 M unpaired light-chain sequences reported	Pretraining/held-out task evaluation; no antigen-level discovery split	N/A	Masked/self-supervised representation learning; paratope prediction metrics	Embedding analyses and paratope-prediction performance reported	No independent target-specific binding validation	COMP	N/A	Not candidate developability	Code/notebooks and deposited resources reported	Useful foundation prior, but paratope prediction and target binding are different claims
AntiBERTy [7]	2021; preprint/used in later work	Antibody language-model representation learning and repertoire analysis	Foundation prior	Antibody sequences	No antigen conditioning	No structure input for pretraining	No; sequence-level antibody LM	Contextual residue/sequence embeddings; repertoire-level analyses	OAS natural antibody sequences	558 M natural antibody sequences reported	Pretraining/evaluation regime; no target-level split applicable	N/A	Masked-language modeling/representation analysis/downstream use	No single cross-method metric; training scale and representation utility are key descriptors	No independent target-specific assay in original AntiBERTy study	COMP	N/A	Representation infrastructure, not candidate developability	Code/model availability reported in associated repository	No target interface; antigen-specific claims depend on downstream task and validation
AbLang [8]	2022; peer reviewed	Antibody language model for completing missing residues	Foundation prior	Heavy-chain or light-chain antibody sequences	No antigen conditioning	No structure input	No; separate heavy- and light-chain models	Residue/sequence representations; masked-residue completion	OAS antibody sequences	OAS-derived heavy/light-chain training data; >40% missing first 15 residues issue reported as use case	Held-out residue-restoration benchmark; no antigen-level split applicable	N/A	Missing-residue restoration; comparison with IMGT germline and ESM-1b	Restoration accuracy/completion performance reported	No independent antigen-binding validation	COMP	N/A	No candidate developability assay	Software/model availability reported	Sequence restoration supports repertoire processing, not target-specific binding or design utility
IgLM [9]	2023; peer reviewed	Generative antibody language modeling and infilling	Foundation prior/generator–optimizer	Antibody sequence with species and chain-type conditioning	No antigen conditioning	No structure input in core LM	No native paired VH/VL generation in original model	Full variable-region sequences; variable-length span infilling including CDRs	Natural antibody sequences	558 M natural antibody sequences reported	Sequence-generation and infilling evaluations; no antigen-level generalization test	N/A	Likelihood/generation quality; diversity; repertoire likeness; *in silico* property analyses	Generation, infilling and antibody-likeness metrics reported	No independent target-specific binding assay in original model	COMP	No experimental attrition	*in silico* aggregation/solubility/humanness-related analyses; no experimental developability	Code/model availability reported	Antibody-like sequence generation does not establish target binding
BALM [12]	2024; peer reviewed	Bio-inspired antibody language model for antibody function and structure prediction	Foundation prior/foundation-prior-derived evaluator	Antibody sequences; antibody-aware positional information	No direct antigen conditioning in pretraining	Structure/function prediction tasks used for evaluation; structure is not the sole input modality	Depends on task; paired-chain handling is task-specific	Antibody representations for downstream prediction tasks	Antibody sequence datasets and downstream functional/structural tasks	Exact task sizes are reported in the primary paper rather than summarized here	Task-specific benchmark splits	N/A or task-specific labels	Function/structure prediction benchmarks	Task-specific prediction metrics reported	No new independent target-specific candidate validation as table evidence	COMP	N/A	No candidate-level developability assay as core evidence	Availability reported in primary article or repository	Downstream predictive utility depends on each benchmark and label regime
AbNatiV [13]	2024; peer reviewed	Antibody/nanobody nativeness assessment for hit selection and humanization	Scorer–ranker/foundation-prior-derived evaluator	Fv or VHH sequence	No antigen conditioning	No explicit target structure input	Assesses sequence nativeness; not paired generation	Nativeness score; humanness/VHH-nativeness assessment	Curated antibody and camelid nanobody sequences	Training/evaluation sets reported in the primary paper	Sequence-level validation; no target-transfer split applicable	N/A	Nativeness scoring against immune-system-derived distributions	Nativeness and hit-selection/humanization utility metrics reported	No independent binding/specificity validation as core table evidence	COMP	N/A	Nativeness/humanness proxy; not experimental immunogenicity or developability	Tool/package availability reported	Nativeness can support triage but does not establish binding or candidate performance
nanoBERT [14]	2024; peer reviewed	Nanobody-specific language modeling/mutation-space navigation	Foundation prior	Nanobody/VHH sequence	No antigen conditioning	No explicit structure input	Not paired; VHH/single-domain context	Residue probabilities/sequence scoring/mutation suggestions	Nanobody sequence corpus	10 million camelid nanobody sequences reported by model page; paper reports domain-specific nanobody LM	Sequence-level residue-prediction benchmark	N/A	Amino-acid prediction; comparison with human-based LMs and ESM-2	Residue-prediction performance reported	No independent antigen-binding validation	COMP	N/A	No candidate developability assay as core evidence	Availability reported through publication/model resources	Domain-specific sequence scoring is not target-specific binding evidence
p-IgGen [15]	2024; peer reviewed	Paired antibody generative language modeling	Foundation prior/generator–optimizer	Unpaired and paired antibody sequences	No antigen conditioning	No structure input as conditioning; 3D/developability descriptors used in evaluation or fine-tuning context	Yes; paired VH/VL generation	Paired heavy–light variable-region sequences	OAS-derived unpaired and paired VH/VL data	1.8 M paired VH/VL sequences reported for p-IgGen fine-tuning	Sequence-generation evaluation; paired zero-shot overlap checks reported	N/A	Diversity, novelty, antibody-like properties, pairing realism and computational developability descriptors	Paired generation and property-distribution metrics reported	No independent antigen-specific binding assay in primary study	COMP	No wet-lab attrition	Predicted/structure-derived developability descriptors; not experimental developability	Article and preprint resources reported	Paired realism and predicted developability do not establish target-specific function
IgBert/IgT5 [10]	2024; peer reviewed	Large-scale paired and unpaired antibody language modeling	Foundation prior	Paired and unpaired antibody variable-region sequences	No direct antigen conditioning	No structure input in core pretraining	Yes; models handle paired and unpaired variable-region inputs	Antibody embeddings; sequence recovery and downstream prediction inputs	Observed Antibody Space paired and unpaired sequences	Over 2B unpaired and 2 M paired antibody sequences reported	Task-specific benchmark evaluation; no antigen-level discovery split as core pretraining evaluation	N/A	Sequence recovery, expression and binding-affinity prediction tasks	State-of-the-art performance on reported antibody-engineering tasks	No new independent candidate wet-lab validation as core evidence	COMP	N/A	Downstream expression/binding prediction tasks; not experimental developability validation by itself	Publication and code/resources reported	Strong foundation prior; downstream claims depend on task labels and evaluation regime
ATUE/EATLM [16]	2023; preprint/benchmark-resource context	Benchmarking pre-trained language models for antibody tasks	Benchmark/foundation-prior evaluator	Antibody sequences; task-specific labels	Varies by task; benchmark spans different specificity levels	No unified structure input in benchmark definition	Depends on task	Task performance across antibody understanding/evaluation tasks	ATUE benchmark datasets	Four antibody prediction tasks reported in ATUE description	Benchmark-specific splits	Task-specific; not a single negative-construction protocol	Multi-task benchmark metrics	Performance across PLMs and antibody tasks reported	No new independent validation; uses benchmark labels	COMP	N/A	Not a candidate developability assay	ATUE and code released according to project/repository	Benchmark supports representation comparison but is not itself discovery validation
Mason *et al.* [17]	2021/2023; peer reviewed article available	Target-specific antibody optimization by sequence-based specificity prediction	Scorer–ranker/generator–optimizer workflow	Trastuzumab variant sequences	Implicit; HER2 context enters through experimental specificity labels	No direct antigen structure input to predictive model	Original target scaffold context; not a general paired-generation model	Candidate-level HER2 specificity score/ranked variants	Mammalian-displayed trastuzumab variant libraries and prospective candidate testing	Large experimental screening and computational variant search reported in source article	Within-program target-specific training/testing and candidate selection; not antigen-holdout transfer	N/A; experimentally labeled variants rather than synthetic Ab–Ag negatives	Predictive performance and prospective candidate testing	Affinity/specificity retention and selected-variant testing outcomes reported	Experimental testing of selected predicted variants	COMP + SPEC	Partial; selected/tested candidates reported	Expression/testing reported; broader developability not comprehensive	Availability not clearly reported in extracted table source	Strong within-target optimization but not a general unseen-antigen predictor
Hie *et al.* general protein LM-guided antibody evolution [18]	2024; peer reviewed	Protein-language-model-guided antibody mutation/evolution	Generator–optimizer/scorer-guided mutational proposal	Antibody sequence; general protein LM mutation suggestions	No antigen input to language model; target effect assessed through assays	No structure input as core model conditioning	Applies to antibody sequences; paired-chain details are case-specific	Suggested mutations/evolved antibody variants	Human antibody optimization case studies	Case-specific variant numbers reported in primary paper	Case-study experimental evaluation rather than standardized split benchmark	N/A	Experimental evolution/optimization outcomes; task-specific assays	Binding/fitness improvement outcomes reported in source article	Experimental testing reported	COMP + BIO	Attrition/variant testing details reported at case-study level	Functional assay evidence; broader developability status depends on case	Availability not clearly reported in extracted table source	General PLM plausibility lacks explicit target conditioning; assay evidence is case-specific
AbAgIntPre [19]	2022; peer reviewed	Sequence-based antibody–antigen interaction prediction	Scorer–ranker	Antibody and antigen amino-acid sequences	Explicit antibody–antigen sequence conditioning	No structure input	Not a paired VH/VL generative model	Pair-level interaction probability	SAbDab-derived antibody–antigen complexes with constructed positive/negative pairs	Dataset construction reported in primary paper and repository	Antigen/subgroup-based and independent-test evaluation reported; biological holdout details should be read from source protocol	Synthetic negatives constructed by pairing rules; exact protocol is source-defined	Binary classification; AUROC/AUC and related metrics	AUC/AUROC and classification metrics reported	No prospective experimental validation	COMP	No wet-lab attrition	No experimental developability	Repository/web resources reported	Performance depends on constructed negative regime and split/generalization unit
Sardar *et al.* nanobody–antigen prediction [20]	2023; conference/arXiv	Nanobody–antigen binding prediction from sequence	Scorer–ranker	Nanobody and antigen sequences/gapped *k*-mer features	Explicit nanobody–antigen sequence conditioning	No structure input	N/A; nanobody single-domain	Pair-level binding classification	Curated nanobody–antigen binding and nonbinding data	Dataset size reported in source paper	Repeated random train–test partitioning reported	Nonbinding examples constructed by antigen exchange/sequence-distance rules in source protocol	Binary classification; accuracy and related metrics	Up to approximately 90% accuracy reported in abstract/source summary	No prospective experimental validation	COMP	No wet-lab attrition	No experimental developability	Availability not clearly reported in extracted table source	Random partition and synthetic negatives limit target-transfer interpretation
ProtAttBA [21]	2025; peer reviewed	Sequence-only prediction of antibody–antigen binding-affinity changes	Scorer–ranker	Antibody–antigen complex sequences	Explicit antibody–antigen sequence conditioning	No required complex structure input; sequence-only model	Depends on complex sequence input; not generative paired-chain model	Predicted affinity change/ΔΔG-like regression output	Open antibody–antigen affinity-change benchmarks	Three open benchmarks reported	Benchmark-specific splits	N/A or benchmark-derived; not a binary negative-pair task	Regression/correlation on affinity-change benchmarks; robustness/interpretability analyses	Benchmark correlations and regression metrics reported	No new independent experimental validation as table evidence	COMP	N/A	No experimental developability	GitHub reported	Sequence-only robustness is benchmark-dependent; predicted ΔΔG is computational evidence
NABP-BERT [22]	2025; peer reviewed	Nanobody–antigen binding prediction using BERT architecture	Scorer–ranker	Nanobody and antigen sequences	Explicit nanobody–antigen sequence conditioning	No structure input	N/A; nanobody context	Pair-level binding prediction	Nanobody–antigen sequence datasets	Dataset counts reported in source article	Pair-level partitioning in processed dataset	Binding/nonbinding labels and negative construction defined in source protocol	Binary classification; AUROC/accuracy and related metrics	Classification metrics reported	No prospective experimental validation	COMP	No wet-lab attrition	No experimental developability	GitHub reported	Limited target diversity and split design may constrain target-transfer interpretation
RLEAAI [23]	2025; peer reviewed	AAI prediction with PLM features and sequence-order extraction	Scorer–ranker	Antibody and antigen sequences; ESM2-derived embeddings plus sequence-order features	Explicit antibody–antigen sequence conditioning	No direct structure input reported as core modality	Not generative paired-chain modeling	Pair-level interaction prediction	Two antibody–antigen interaction datasets reported	Dataset sizes reported in primary paper	Dataset-specific splits	Benchmark labels; negative protocol is source-defined	AAI prediction metrics; comparison with state of the art	AAI classification metrics reported	No prospective experimental validation as table evidence	COMP	No wet-lab attrition	No experimental developability	Availability not clearly reported in extracted table source	Improved sequence-order modeling does not remove split/negative-regime dependence
DeepInterAware [24]	2025; peer reviewed	Interaction-interface-aware AAI prediction from sequence data	Scorer–ranker	Antibody and antigen sequences; learned interface-aware representations	Explicit antibody–antigen sequence conditioning	Structural interface is modeled through learned/interface-aware design; no experimental structure input required for all cases	Not a paired-chain generator	AAI prediction/candidate interaction score	AAI datasets reported in Advanced Science article	Dataset sizes reported in source article	Unseen antigen/antibody inductive settings emphasized in source article	Benchmark labels/negative construction defined in source protocol	AAI prediction metrics and inductive generalization evaluation	Performance metrics reported for AAI prediction from sequence data	No prospective experimental validation as table evidence	COMP	No wet-lab attrition	No experimental developability	Availability not clearly reported in extracted table source	Interface-aware prediction remains computational unless supported by prospective assays
MambaAAI [25]	2025; peer reviewed	Mamba-based antibody–antigen interaction prediction and binding-site identification	Scorer–ranker	Antibody and antigen sequences represented with ESM-2 features	Explicit antibody–antigen sequence conditioning	No experimental structure input; residue-level interaction matrices used	Not a paired-chain generator	AAI prediction; paratope/epitope-related binding-site identification	Two large-scale antibody–antigen neutralization datasets reported	Dataset sizes reported in source article	Dataset-specific *in silico* evaluation	Benchmark labels/negative construction defined in source protocol	AAI prediction and binding-site identification metrics	*in silico* AAI performance metrics reported	No prospective experimental validation as table evidence	COMP	No wet-lab attrition	No experimental developability	Publication and PMC/PubMed record available	Modern sequence architecture does not by itself establish experimental binding
AbAgIPA [26]	2024; peer reviewed	AAI prediction with backbone-aware invariant point attention	Scorer–ranker	Antibody/antigen with backbone-aware or predicted structural features	Explicit antibody–antigen conditioning	Yes; backbone-aware invariant point attention/structural features	Not a paired-chain generative model	Pair-level interaction prediction	AAI dataset with antigen grouping	Dataset counts reported in primary article	Highly similar antigens grouped; nonoverlapping antigen subsets between training and test data reported	AAI labels and negatives defined in source protocol	Binary interaction prediction; AUROC/accuracy and benchmark comparison	AAI performance metrics reported	No prospective experimental validation	COMP	No wet-lab attrition	No experimental developability	Availability not clearly reported in extracted table source	Stronger holdout improves audit value but remains computational evidence
MuLAAIP [27]	2025; preprint/repository reported	Multi-modality representation learning for AAI prediction	Scorer–ranker	Antibody sequence and structural representations	Explicit antibody–antigen interaction prediction setting	Yes; integrates 3D structural and 1D sequence information	Not a paired-chain generator	Pair-level AAI prediction	Curated AAI benchmark dataset with structural and sequence information	Dataset details reported in preprint	Benchmark-specific evaluation	Benchmark labels/negative construction defined by authors	AAI prediction metrics	Outperformance over reported baselines claimed in preprint	No prospective experimental validation as table evidence	COMP	No wet-lab attrition	No experimental developability	Code/dataset reported as available	Multimodal benchmark is computational and depends on curated-label regime
AsEP/WALLE [28]	2024; NeurIPS Datasets and Benchmarks	Antibody-specific epitope prediction benchmark and model	Benchmark/scorer–ranker	Antibody–antigen complex structure representations plus sequence/PLM features	Explicit antibody–antigen/epitope context	Yes; graph representations of antibody–antigen complexes	Not paired-chain generation	Epitope prediction/antigen surface residue links	Filtered antibody–antigen complex structure dataset	Dataset size and filtering reported in benchmark paper	Random and epitope-group generalization settings reported	N/A; epitope labels derived from complexes	Epitope prediction metrics; WALLE versus protein-binding-site baselines	3–10× improvement over baselines reported in source summary	No new prospective wet-lab validation	COMP	N/A	No candidate developability	Data/code open source reported	Epitope prediction benchmark, not candidate binding validation
AbBiBench [29]	2025; preprint/benchmark	Benchmark for antibody binding-affinity maturation and design	Benchmark/evaluation framework	Antibody sequences and Ab–Ag complex context depending on evaluated model	Task-specific; evaluates antibody designs with antigen context	Yes; evaluates Ab–Ag complex as functional unit and includes structures	Heavy-chain mutated antibodies; paired-chain status per dataset	Affinity-oriented evaluation and generation/ranking over antibody variants	Curated experimental affinity datasets across antigens	9 datasets, 9 antigens and 155,853 heavy-chain mutated antibodies reported	Dataset/antigen-specific benchmark; zero-shot/unseen antigen settings reported	N/A; affinity regression/ranking rather than constructed binary negatives	Correlation between model likelihood and experimental affinity; generation/ranking tasks	Correlation and task-specific outcomes reported	*in vitro* ELISA binding assay reported for generation case study	COMP + BIO	Partial; selected/generated candidate denominator reported in case study	Structural integrity and biophysical properties used in generation ranking; mostly computational	Code/data reported on GitHub/HuggingFace/Zenodo	Recent preprint benchmark; no single metric should be pooled across all tasks
AVIDa-SARS-CoV-2/VHHCorpus-2 M/VHHBERT [30]	2024; preprint/dataset-resource context	SARS-CoV-2 VHH interaction dataset and nanobody LM benchmark	Benchmark/foundation prior/scorer–ranker	VHH sequences and SARS-CoV-2 antigen variants	Explicit antigen/VHH binding benchmark	No required structure in core source abstract	N/A; VHH/single-domain	Binding prediction/VHH language-model evaluation	AVIDa-SARS-CoV-2 interaction dataset; VHHCorpus-2 M pretraining corpus	VHHCorpus-2 M contains over 2 M VHH sequences according to source context	Task-specific split based on antigen variants reported in benchmark context	Binary binding/nonbinding labels from dataset protocol	Binding prediction benchmark; LM representation evaluation	Task-specific performance metrics reported	No new prospective validation as table evidence	COMP	N/A	No experimental developability as core evidence	Dataset/model resources reported	SARS-CoV-2/VHH-specific benchmark may not generalize to broad antibody discovery
DiffAb [31]	2022; peer reviewed/preprint lineage	Antigen-conditioned antibody CDR sequence–structure generation	Generator–optimizer	Antibody–antigen structure/geometry context	Explicit structure-conditioned antigen context	Yes; antigen–antibody geometric conditioning	Generates antibody CDRs within structural context; not full paired VH/VL discovery platform	CDR sequences and structures	SAbDab antibody–antigen structures	Dataset size/splits reported in paper	Structure-based benchmark splits; generalization depends on structural dataset partitioning	N/A	Amino-acid recovery, RMSD, docking/energy/proxy metrics	Computational design and structure metrics reported	No independent wet-lab binding validation as core evidence	COMP	No experimental attrition	Computational energy/biophysical proxies only	Code/resource availability reported	Structure-conditioned design metrics are not equivalent to independent binding evidence
MEAN [32]	2022; ICLR/arXiv	Conditional antibody CDR sequence–structure co-design	Generator–optimizer	Antibody–antigen complex graph with target antigen and antibody light-chain context	Explicit antigen and antibody-context conditioning	Yes; E(3)-equivariant graph translation over complex context	Uses light-chain context; not paired VH/VL generation from scratch	CDR 1D sequence and 3D structure	Antibody–antigen structural datasets	Dataset details reported in paper/repository	Benchmark splits reported by paper	N/A	Sequence recovery, structural RMSD, antigen-binding CDR design and affinity-optimization benchmarks	Relative improvements over baselines reported in abstract/source	No independent wet-lab validation as core table evidence	COMP	No experimental attrition	Computational structure/affinity proxies only	Code reported in repository	Co-design/recovery metrics do not by themselves establish functional antibody discovery
dyMEAN [33]	2023; peer reviewed/conference	Full-atom antibody design with equivariant generative modeling	Generator–optimizer	Antibody–antigen structural context	Explicit antigen/complex structural conditioning	Yes; full-atom structural design	Not a paired VH/VL sequence-only LM; structural complex context	Antibody sequences and structures, especially CDR design	SAbDab-derived antibody–antigen structures	Dataset size and CDR-H3 clustering protocol reported in paper	CDR-H3 cluster-aware partitioning reported	N/A	Amino-acid recovery, RMSD and related structural/generative metrics	Recovery and structure metrics reported; terminal-position effects discussed in benchmarks/literature	No independent wet-lab validation as core table evidence	COMP	No experimental attrition	Computational developability/structure metrics only	Code/resource availability reported	Recovery can measure reconstruction under dataset bias rather than functional design
IgDiff [34]	2024/2025; preprint and journal article	Antibody variable-domain SE(3) diffusion model	Generator–optimizer	Antibody variable-domain structural context	No explicit antigen conditioning in core variable-domain diffusion tasks	Yes; SE(3) structural diffusion over antibody domains	Can handle multiple chains in variable-domain context; not target-conditioned paired discovery	Antibody variable-domain backbones/designs	Antibody structure datasets	Dataset details reported in paper	Structural generation benchmarks	N/A	Designability, novelty, dihedral agreement and antibody-design task metrics	Designability/novelty metrics reported	Expression of designed antibodies experimentally verified; binding not necessarily target-specific	COMP + DEV	Partial; expression-tested designs reported	Expression yield experimentally assessed; broader developability limited	Availability not clearly reported in extracted table source	Expression validates production feasibility, not antigen-specific binding
AB-Gen [35]	2023; peer reviewed/preprint context	Predictor-guided CDR-H3 generation for target-specific antibody design	Generator–optimizer	Antibody sequence; target-specific predictor/reward context	Implicit target conditioning through predictor/objective rather than direct antigen input	No direct antigen structure input as core generator modality	Not paired VH/VL generation	CDR-H3 sequences	Repertoire/antibody sequences and target-specific predictors	Data and predictor details reported in source	Computational generation and predictor-based evaluation	N/A	Validity/diversity/novelty and predictor-derived binding/specificity objectives	Computational proxy improvements reported	No independent experimental validation as core table evidence	COMP	No wet-lab attrition	Predicted properties only	Availability not clearly reported in extracted table source	Optimizing a predictor does not equal measured binding or specificity
PALM-H3 [36]	2024; peer reviewed	De novo generation of SARS-CoV-2 antibody CDR-H3 with PLM guidance	Generator–optimizer	Antibody sequence plus SARS-CoV-2 antigen/task context	Explicit or task-specific antigen context for SARS-CoV-2 spike design	Structural analyses used; core model is sequence/generation oriented	Seed/scaffold context; not full paired VH/VL generation from scratch	CDR-H3 sequences	SARS-CoV-2 antibody/antigen-specific training and generation data	Data and candidate numbers reported in primary paper	Task-specific generation and validation rather than broad split benchmark	N/A	Generation quality, binding assays and neutralization-related outcomes	*in vitro* binding and neutralization evidence reported	ELISA/binding and neutralization assays for generated antibodies	COMP + BIO	Partial; candidates selected/tested reported in paper	Functional assays; broader developability not comprehensive	Availability not clearly reported in extracted table source	CDR-H3-only output leaves scaffold and paired-chain compatibility to workflow
BetterBodies [37]	2024; preprint	RL-guided latent diffusion for antibody CDR-H3 sequence design	Generator–optimizer	CDR-H3 sequences; simulator/reward-derived target signal	Implicit target conditioning through Absolut! simulator reward	No real structural input; simulator defines synthetic binding environment	No; CDR-H3-only	Novel CDR-H3 sequences	Sequence data and Absolut! simulator-generated evaluations	Absolut! simulator and dataset details reported	*in silico* simulator benchmark	N/A; simulator labels/rewards	Novelty/diversity and simulator-derived affinity/biophysical objectives	Improved simulator affinity to SARS-CoV spike RBD reported	No independent experimental validation	COMP	No wet-lab attrition	Simulated/predicted biophysical properties only	Availability not clearly reported in extracted table source	Simulator success is not measured binding or candidate usability
AntiBARTy Diffusion [38]	2024/2025; preprint/method context	Antibody sequence generation with diffusion/sequence modeling	Generator–optimizer	Antibody sequence	No direct antigen conditioning in core generation	No required structure input in core sequence generation	Usually chain/sequence generation; paired status source-specific	Antibody sequences/CDRs depending on task	Antibody sequence datasets	Data scale reported in source	Sequence-generation benchmark	N/A	Generation quality, validity, novelty/diversity and antibody-likeness metrics	Computational generation metrics reported	No independent experimental validation as core table evidence	COMP	No wet-lab attrition	Predicted properties only if reported	Availability not clearly reported in extracted table source	Sequence novelty and antibody-likeness do not establish target-specific function
MAGE [39]	2025; peer reviewed/PMC	Antigen-specific paired-chain antibody generation using LLMs	Generator–optimizer	Antigen sequence/context and paired antibody variable-region generation	Explicit target/antigen-conditioned generation reported	No structure input as central generator modality; sequence-based PLM	Yes; paired heavy-light chain antibodies	Paired human VH/VL antibody sequences targeting input antigen contexts	Training data and target-specific generation data reported in article	Data and candidate counts reported in primary paper	Task-specific experimental validation rather than standardized split benchmark	N/A	Diversity/novelty and antigen-specific binding validation	Experimental binding/specificity results reported for generated antibodies	Experimental antigen-specific binding assays reported	COMP + BIO + SPEC	Partial; generated/selected/tested counts reported in article	Candidate properties assessed to the extent reported; broader developability limited	Publication and resources reported	Experimental success is target/workflow-specific and not a general benchmark score
Germinal [40]	2025; preprint/article resource	Epitope-targeted de novo antibody design framework	Generator–optimizer	Target protein and specified epitope context; sequence/structure co-optimization	Explicit epitope-targeted conditioning	Yes; integrates structure prediction/filtering with sequence generation	Generates antibody candidates; paired-chain format depends on design protocol	De novo antibodies against specified epitopes	Target-specific design campaigns	Four targets and low-throughput validation campaign reported in source summaries	Campaign-based prospective validation rather than standardized split benchmark	N/A	Experimental screening and BLI/affinity measurement after computational design	Nanomolar binders and target-level success rates reported in source	Split-luciferase screening and BLI/kinetics reported	COMP + BIO + SPEC	Partial; low-throughput tested-candidate counts/success rates reported in source	Some candidate properties measured; full developability package not comprehensive	Code/protocol openness reported by source summaries	Strong experimental evidence, but recent and campaign-specific
RFdiffusion/RFAntibody [41]	2025; peer reviewed/Nature context	Atomically accurate de novo antibody design with target/epitope structural conditioning	Generator–optimizer	Target structure and specified epitope with antibody design model	Explicit structure/epitope conditioning	Yes; all-atom/structural design context	Generates antibody binders; paired-chain details are design-format dependent	De novo antibodies/antibody binders	Target-specific computational design campaigns with experimental testing	Campaign details and success rates reported in primary Nature article	Prospective design/testing; not a reusable benchmark split	N/A	Experimental binding and structural agreement for selected designs	Experimental binding and atomic-level structural validation reported	Independent binding assays and experimental structures reported for selected binders	COMP + BIO + STR	Partial; design-to-test attrition reported in paper	Developability assessment limited to reported candidate assays	Availability not clearly reported in extracted table source	High evidence for selected cases, but campaign-specific success rates are not directly comparable to benchmark metrics
tFold system [42]	2025; peer reviewed	Structure-driven de novo epitope-specific antibody design workflow	Generator–optimizer/scorer–ranker workflow	Antigen sequence, specified epitope and antibody framework templates	Explicit epitope/antigen conditioning	Yes; structure-guided filtering and antibody–antigen complex prediction	Uses heavy/light framework templates and generates paired Fab designs	CDR-filled Fab/antibody candidates for specified epitopes	Four target design campaigns: Flu A, PD-1, PD-L1 and SC2RBD	10 000 Fab pairs per target synthesized after filtering reported	Prospective campaign validation; no standardized ML split	N/A	SPR KD, competition assays, neutralization/functional assays and novelty analyses	Binder success rate approximately 1.5–4.4%; top KD values reported including 45 pM PD-L1, 3.22 nM Flu A, 2.0 nM SC2RBD and 81.6 nM PD-1	Phage display selection, SPR affinity and SPR competition assays reported	COMP + ENR + BIO + SPEC	Yes; generated/filtered/synthesized and success-rate information reported	Framework chosen for stability/expression/aggregation advantages; broader developability limited	Source article available; software availability not clearly reported in this table	Strong workflow evidence but includes experimental selection/filtering; model confidence alone is insufficient
IgCraft [43]	2025; preprint/open resources	Versatile paired human antibody sequence generation using Bayesian Flow Networks	Foundation prior/generator–optimizer	Paired human antibody sequences with optional conditional tasks	No direct antigen conditioning in core framework	Can condition on structural data for inverse-folding/scaffolding tasks; not primarily target-binding conditioned	Yes; paired human antibody generation	Unconditional sampling, sequence inpainting, inverse folding and CDR motif scaffolding	Paired human antibody sequence data and task-specific benchmarks	Data and task sizes reported in preprint	Task-specific generation benchmarks	N/A	Humanness, structural-property preservation and task-specific generation metrics	Competitive results and state-of-the-art CDR motif scaffolding claimed	No independent target-specific binding validation as core evidence	COMP	No wet-lab attrition	Humanness/structural-property proxies; no broad experimental developability	Model code and weights reported as public	Unified paired generator, but not direct evidence of antigen-specific binding
AntibodyDesignBFN [44]	2026; preprint	Fixed-backbone antibody inverse folding with Discrete Bayesian Flow Networks	Generator–optimizer	Fixed 3D antibody backbone/geometric constraints	No explicit antigen conditioning in core fixed-backbone inverse-folding task	Yes; Geometric Transformer with IPA and fixed-backbone conditioning	Designs sequences for antibody backbones; paired-chain status task-dependent	Antibody sequences conditioned on fixed backbone	SAbDab/antibody structural data and 2025 temporal test set reported	2025 temporal test set reported	Temporal test set used for evaluation	N/A	Amino-acid recovery and inverse-folding metrics	48.1% H-CDR3 AAR reported on rigorous 2025 temporal test set	No independent experimental validation as table evidence	COMP	No wet-lab attrition	No experimental developability	Code and checkpoints reported on GitHub/HuggingFace	High-fidelity inverse folding is not equivalent to measured antigen binding
AgForce [45]	2026; preprint	Antigen-conditioned CDR sequence–structure co-design model	Generator–optimizer	Antigen/antibody graph context for CDR sequence–structure co-design	Explicit antigen-conditioned design	Yes; GNN encoder, hyperbolic cross-attention and sequence–structure co-design components	Not full paired-chain discovery; CDR-focused design context	Antigen-conditioned CDR sequence–structure designs	CHIMERA-Bench dataset	CHIMERA-Bench 2922 complexes used as benchmark context	CHIMERA-Bench split settings: unseen epitopes, unseen antigen folds and temporal targets	N/A	Binding quality, sequence recovery and interface metrics on CHIMERA-Bench	8% AAR improvement over strongest sequence baseline and improved interface metrics claimed in abstract	No independent wet-lab validation as table evidence	COMP	No wet-lab attrition	No experimental developability	Source code reported at GitHub	Addresses antigen-blindness computationally; still requires experimental validation
AbMEGD [46]	2025; IJCAI/arXiv	Multi-scale equivariant graph diffusion for antibody sequence–structure co-design and optimization	Generator–optimizer	Atomic-level geometric features plus residue-level embeddings in Ab–Ag context	Explicit antigen/complex design setting	Yes; multi-scale E(3)-equivariant graph diffusion	Not a paired-chain sequence LM; structural co-design context	Antibody sequence and structure co-design; optimization for complex antigen binding	SAbDab data collected up to September 2024 reported	Dataset filtering and benchmark details reported in IJCAI paper	Benchmark splits reported in source paper	N/A	AAR, RMSD and improvement percentage compared with DiffAb and baselines	10.13% AAR increase, 3.32% improvement-percentage rise and 0.062 Å CDR-H3 RMSD reduction versus DiffAb reported in abstract/source	No independent wet-lab validation as table evidence	COMP	No wet-lab attrition	Computational structural/function proxies only	Code reported as available	Structure/co-design improvements remain computational without assay validation
Origin-1 [47]	2026; bioRxiv/platform preprint	Generative AI platform for de novo antibody design against zero-prior epitopes	Generator–optimizer	Target/epitope context for de novo antibody design	Explicit epitope/target conditioning	All-atom/structural modeling described by platform source; exact model internals require source-specific reading	Generates full antibody candidates; format depends on campaign	De novo antibodies against zero-prior epitopes	Ten zero-prior human protein epitope campaigns reported by platform source	Successful targets reported for four of ten campaigns by platform source	Prospective campaign validation; not a standardized benchmark split	N/A	Binding/orthogonal assay validation and target-level success metrics	Platform source reports success for four targets; exact assay/attrition details should be read from preprint	Experimental validation reported by platform/preprint source	COMP + BIO	Partial; campaign-level success information reported	Developability/orthogonal assays reported by source but require detailed extraction before final citation	bioRxiv/platform source available	Commercial/platform preprint; method details and reproducibility may be limited
CHIMERA-Bench [48]	2026; preprint/benchmark	Benchmark dataset for epitope-specific antibody design	Benchmark/evaluation framework	Antibody–antigen complexes with epitope/paratope annotations	Explicit epitope-conditioned design benchmark	Yes; complex structures and interface annotations	CDR-focused design benchmark; paired-chain handling follows complex data	Evaluation protocol for epitope-conditioned CDR sequence–structure co-design	Curated/deduplicated antibody–antigen complex dataset	2922 antibody–antigen complexes reported	Three biologically motivated splits: unseen epitopes, unseen antigen folds and prospective temporal targets	N/A	Five metric groups including epitope-specificity measures	Representative methods benchmarked across all splits	No new wet-lab validation; benchmark uses structural/computational labels	COMP	N/A	No experimental developability	Code/data reported at GitHub	Benchmark improves comparability but remains computational and recent preprint
NbBench [49]	2025; preprint/benchmark	Benchmarking language models for nanobody engineering tasks	Benchmark/evaluation framework	Nanobody/VHH sequences and task-specific labels	Task-dependent; not a universal antigen-conditioned benchmark	No universal structure input; task-specific features may vary	N/A; nanobody single-domain	Nanobody LM evaluation across engineering tasks	Nanobody sequence/task datasets	Dataset/task details reported in source	Task-specific splits	Task-specific labels	Language-model benchmark metrics for nanobody tasks	Task-specific performance metrics reported	No new prospective wet-lab validation as table evidence	COMP	N/A	Some tasks relate to thermostability/engineering labels; experimental status depends on dataset	Source resources reported	Nanobody-specific benchmark; transfer to conventional VH/VL antibodies is limited
NanoAbLLaMA [50]	2025; peer reviewed	Nanobody library construction using a nanobody large language model	Foundation prior/generator–optimizer	Nanobody/VHH sequences	No direct antigen conditioning in core library construction	No explicit structure input as core generator modality	N/A; nanobody single-domain	Synthetic nanobody library sequences	Nanobody sequence data and library-design tasks	Dataset and library details reported in article	Generation/library evaluation rather than antigen-level split benchmark	N/A	Library quality, germline-specific design and sequence-diversity metrics	Library-generation metrics reported	No independent target-specific binding validation as core evidence	COMP	No wet-lab attrition	Library-level sequence/design properties; not broad experimental developability	Article available in PMC	Useful nanobody-generation case, but not direct target-specific candidate validation
S2ALM [51]	2025; preprint/article context	Sequence–structure multi-level pre-trained antibody language model	Foundation prior	Antibody/protein sequence plus 3Di structural tokens	No direct antigen conditioning in pretraining	Yes; sequence–structure pretraining via 3Di/Foldseek-style structural encoding	Not a paired-chain generator by itself	Multi-level antibody representations for downstream tasks	Large-scale sequence and structure corpora reported in source	Large-scale antibody/protein sequence-structure data reported	Pretraining and downstream benchmark evaluations	N/A	Representation learning, sequence–structure matching and downstream tasks	Task-specific performance metrics reported	No prospective experimental candidate validation as table evidence	COMP	N/A	Not candidate developability validation	Availability not clearly reported in extracted table source	Hybrid sequence–structure representations are useful but remain benchmark-dependent

## Coordinate system

Most included methods can be organized into a three-layer functional stack which could describe a method’s functional role in a discovery workflow: (i) foundation priors, (ii) scorer–rankers, and (iii) generator–optimizers. Foundation priors learn reusable representations or generative distributions from large antibody-sequence corpora and provide embeddings, likelihoods, or pretrained generation capabilities for downstream use. Scorer–rankers map antibody candidates, with explicit or implicit target context where applicable, to prediction or prioritization scores. Generator–optimizers propose or edit antibody sequences under conditioning signals, learned objectives, or external optimization criteria.

To interpret a reported capability, we additionally use three comparison axes which provide a compact audit scaffold that can be applied across foundation priors, scorer–rankers, and generator–optimizers. Other descriptors, including architecture, model scale, data volume, modality integration, and computational cost, remain important, but differences in conditioning, output, and validation most directly change how generalization, molecular utility, and empirical support should be interpreted. Section 4.4 illustrates this interpretive role through representative benchmark comparisons.

The conditioning interface records how target context enters a method. We distinguish antibody-only methods; implicit target conditioning through target-specific labels, rewards, or external oracles; explicit antibody–antigen sequence conditioning; epitope- or peptide-level conditioning; and structure-, surface-, or multimodal conditioning. The distinction concerns target exposure rather than a hierarchy of model quality. For example, AbAgIntPre, and NABP-BERT use antibody–antigen sequence pairs, PALM-H3, and Germinal use explicit epitope context, and structure-conditioned methods such as DiffAb provide geometric target information while retaining antibody sequence as a designed object [19, 22, 31, 36, 40]. A method can therefore be strongly optimized for a target without receiving the target itself as a direct model input.

Output granularity records the molecular or decision object produced by a method. Scorer–rankers may return binary interaction predictions, mutation-effect estimates, or continuous affinity-related scores. Generative outputs range from CDR-H3-only designs to multi-CDR or joint sequence–structure outputs, full or near-full variable regions and paired VH/VL sequences. Moving toward more complete outputs increases molecular commitment: fewer aspects of the final candidate are delegated to a fixed scaffold or downstream pipeline, but compatibility, folding, pairing, and candidate-level properties become increasingly relevant to evaluation. p-IgGen and Monoclonal Antibody Generator (MAGE), for example, illustrate paired VH/VL outputs, whereas AB-Gen and BetterBodies restrict generation to CDR-H3 [15, 35, 37, 39]. Output granularity therefore describes commitment, not model quality or experimental evidence.

For annotation in this review, the validation evidence profile separates training supervision, internal model evaluation, and independent validation. Experimental measurements used only as labels, rewards, or oracle signals remain part of the supervision or optimization process; held-out or cross-validation performance remains computational/internal evidence even when the reference labels were experimentally derived. We use six nonordinal evidence categories: COMP (computational/internal), ENR (independent enrichment or selection), BIO (independent quantitative binding or biophysical), SPEC (independent specificity mapping), STR (experimentally resolved structure, or complex), and DEV (experimental developability). A study may receive multiple labels.

COMP includes held-out discrimination or regression metrics, likelihood-based evaluations, predicted affinity or mutation effects, computational energy functions, docking, predicted antibody–antigen complexes, and *in silico* developability scores. ENR denotes experimentally observed selection or enrichment on validation candidates not used to fit or directly optimize the model; training-label enrichment or predicted enrichment does not qualify as independent ENR evidence. BIO denotes direct quantitative experimental measurements of binding strength or kinetics on independent validation candidates, including equilibrium or kinetic measurements by SPR or BLI and experimentally derived titration-based affinity estimates. Predicted affinity, ΔΔG, computational free-energy scores, enrichment ratios, and binary binding labels do not by themselves qualify. SPEC denotes an experimental design capable of distinguishing among relevant antigens, epitopes, or competitors. Positive binding to a single target is insufficient for a specificity label, and predicted specificity remains COMP unless independently tested on an appropriate panel. STR denotes experimental determination of the relevant antibody–antigen complex or binding interface, for example, by X-ray crystallography or cryo-electron microscopy. Docking, molecular dynamics, energy-minimized models, and predicted complexes remain computational structural hypotheses unless compared with an independent experimentally determined structure. DEV denotes direct experimental measurement of the developability properties claimed for model-selected or generated candidates, such as expression, stability, solubility, aggregation, viscosity, or polyspecificity; computational developability scores remain COMP.

These categories form a claim-aligned profile rather than a hierarchy of universally increasing strength. Quantitative binding, specificity, and structural and developability measurements answer different questions and may be complementary within the same study. Cross-study comparison should therefore report which evidence domains are demonstrated and whether they align with the capability being claimed.

**Figure 1:**
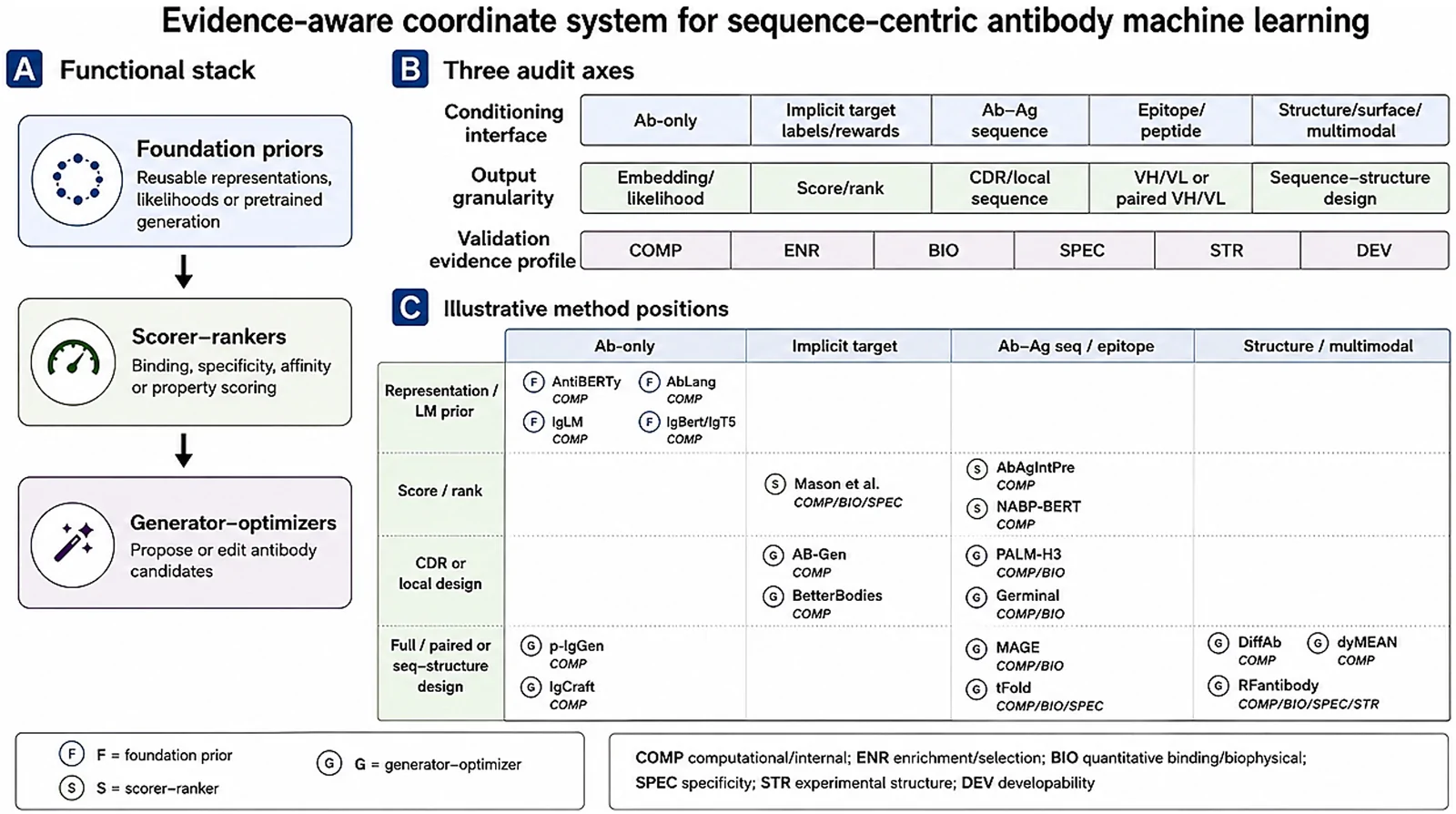
Evidence-aware coordinate system and illustrative mapping of representative sequence-centric antibody machine-learning methods. (A) The functional stack distinguishes foundation priors, scorer–rankers, and generator–optimizers according to their role in an antibody discovery workflow. (B) the three audit axes record target-conditioning interface, molecular output granularity, and validation evidence profile. Evidence domains are nonordinal, and multiple domains may apply to the same study. (C) Selected representative methods from Table 1, including the analytical anchor cases discussed in “Method landscape and representative case studies”, are positioned schematically according to their dominant conditioning regime and output object. Additional examples are included to cover regions of the comparison space that are not fully represented by the anchor cases alone. F, S, and G denote foundation prior, scorer–ranker, and generator–optimizer, respectively. Placements are illustrative and are not intended as a complete inventory or performance ranking.

The overall framework and illustrative positions of representative methods are summarized in Fig. 1.

## Method landscape and representative case studies

The structured literature mapping identified a broader landscape than can be examined method by method in the main text. Table 1 therefore provides a standardized synthesis of the included major methods, covering task and stack layer, input and conditioning modality, structural information, paired-chain modeling, output object, data and scale, split or generalization strategy, negative construction where applicable, benchmark type, reported evaluation metrics and key outcomes, validation evidence profile, independent experimental validation type, failure or attrition reporting, developability assessment, code/model/data availability, and major comparison limitations. The subsections below examine selected analytical anchor cases. Architectural details are retained only when they materially alter conditioning, output, or interpretation of validation.

### AntiBERTy: an antibody-only representation prior

AntiBERTy illustrates the foundation-prior end of the comparison space [7]. It is trained on 558 million nonredundant natural antibody sequences from OAS using masked language modeling and has no antigen or target interface [5]. Under Axis 1, this antibody-only conditioning limits direct claims about antigen-specific recognition: the model learns repertoire regularities rather than an explicit antibody–target relationship. Its primary outputs are contextual residue representations and sequence-derived features, representing a thin output under Axis 2. Ruffolo *et al.* use these representations to examine repertoire organization and affinity-maturation-like trajectories and further apply weak supervision to identify sequence patterns associated with highly redundant repertoire members. The demonstrated evidence profile remains COMP; experimentally derived target-specific validation is not part of the study. AntiBERTy is therefore best interpreted as reusable representation infrastructure. Its principal limitation for discovery-level comparison is not model capacity but the absence of explicit target conditioning, which makes downstream antigen-specific claims dependent on the task and data introduced after pretraining.

### Ablang: antibody-only completion and reusable representations

AbLang provides a second antibody-only foundation prior but commits to a more specific utility: sequence completion [8]. Separate heavy- and light-chain models are trained on nonredundant OAS sequences, and antibody sequence is the only conditioning input [5]. The model outputs residue representations and masked-residue likelihoods; in the original study, these are primarily evaluated by restoring missing sequence regions, particularly systematic N-terminal truncations in repertoire data. This narrower output–task alignment distinguishes AbLang from AntiBERTy: both are target agnostic, but AbLang demonstrates a defined preprocessing utility rather than using embeddings mainly for repertoire-level analysis. Its validation evidence is COMP, based on held-out restoration performance against germline-based imputation and a general protein language–model baseline. No negative-pair construction or antigen-level split is applicable because the task does not model antibody–antigen interaction. The main limitation is consequently one of claim scope: successful sequence restoration and transferable embeddings should not be interpreted as evidence of target-specific binding or design utility without a downstream target–aware evaluation.

### IgLM: antibody-only generative infilling

IgLM moves from representation learning to sequence proposal while retaining an antibody-only conditioning interface [9]. Trained on 558 million nonredundant antibody variable-region sequences, the model generates full variable-region sequences or redesigns variable-length sequence spans through an infilling objective, with species and chain-type metadata providing generation control. Axis 2 is therefore materially thicker than for AntiBERTy or AbLang: IgLM commits to newly generated sequence content, including targeted CDR redesign, rather than merely supplying an embedding or residue likelihood. Evaluation focuses on the diversity, repertoire likeness, and sequence characteristics of generated libraries, and the evidence profile remains COMP. Antigen conditioning, interaction negatives, and antigen-level generalization units are not applicable to the original generation task. The central comparison limitation is that repertoire plausibility is not equivalent to target-specific function. IgLM can generate antibody-like sequence space for subsequent screening, but the original interface and validation do not establish that high-likelihood or diverse generations bind a desired antigen.

### p-IgGen: extending antibody-only generation to paired VH/VL outputs

p-IgGen is an informative Axis 2 comparison because it extends antibody language modeling from individual chains to paired variable regions [15]. The model is pretrained on large unpaired OAS heavy- and light-chain collections and subsequently finetuned on ~1.8 million paired VH/VL sequence. It remains antigen agnostic under Axis 1, but its paired output commits to heavy–light compatibility that is absent from single-chain generation. The original evaluation compares generated pairs with natural paired antibodies using sequence diversity, novelty, antibody-like properties, and structure-derived computational descriptors; paired zero-shot test sequences are reported not to overlap with paired or unpaired training sequences. The evidence profile is COMP. A developability-biased variant is also trained using TAP-predicted structural descriptors, but these predicted properties remain computational guidance rather than experimental DEV evidence. The key strength is pairing-aware molecular output; the corresponding limitation is that paired realism and predicted developability do not by themselves establish antigen-specific binding or experimentally measured candidate properties.

#### Summary (3.1–3.4)

These antibody-only methods occupy a similar conditioning regime but make different molecular commitments. AntiBERTy provides reusable representations, AbLang supports sequence restoration, IgLM proposes variable-region or local sequence redesigns, and p-IgGen generates paired VH/VL candidates. Their original validation profiles are dominated by COMP: naturalness, restoration accuracy, and paired-sequence plausibility support different computational claims, but none independently establishes antigen-specific binding.

### AbAgIntPre: explicit sequence-pair conditioning and interaction scoring

AbAgIntPre marks the transition from antibody priors to explicit target-aware scoring [19]. Its conditioning interface contains both antibody and antigen sequences, and its output is a pair-level probability of interaction. The most consequential methodological choices for comparison are in dataset construction rather than Convolutional Neural Network (CNN) architecture. After redundancy filtering of SAbDab complexes, antigens are grouped by sequence similarity; positive pairs are expanded within defined subgroups, whereas antibodies and antigens from different subgroups are randomly paired to create balanced negative examples [52]. Training and independent test examples are sampled within broader antigen clusters. The evidence profile is COMP because validation remains predictive evaluation on constructed interaction datasets rather than prospective experimental testing. Under Axis 1, the explicit antigen input permits pair-specific scoring, but the negative construction and split unit limit the generalization claim: a model may learn distinctions induced by subgroup construction or antigen-family composition. AbAgIntPre is therefore useful as a triage scorer, while its headline discrimination metrics should be interpreted relative to the constructed negative regime and target holdout design.

### Mason *et al.*: implicit target conditioning through experimental specificity labels

Mason *et al.* provide a contrasting route to target-aware scoring [17]. The predictive model receives trastuzumab variant sequences rather than an explicit HER2 sequence, so target information is introduced implicitly through experimentally generated HER2-specificity labels. The output is a candidate-level specificity score used to rank a computational space of ~10e8 variants. This interface supports powerful optimization within a fixed target and scaffold context but does not, by itself, define an antigen-transfer task. The training data originate from a mammalian-expressed mutational library of ~10e4 trastuzumab variants screened for HER2 specificity. Importantly, the study proceeds beyond internal model performance: recombinant expression and experimental testing of 30 randomly selected predicted variants confirmed retained HER2 specificity. The evidence profile therefore includes SPEC in addition to COMP. Its principal limitation is also its defining strength: target specificity is learned from a target-specific experimental campaign, making the model highly relevant to within-program virtual triage but not directly comparable with explicit antibody–antigen pair models claiming generalization to unseen targets.

#### Summary (3.5–3.6)

AbAgIntPre and Mason *et al.* both rank candidates, but they test different generalization problems. AbAgIntPre explicitly scores antibody–antigen sequence pairs under a constructed multi-antigen dataset, whereas Mason *et al.* learn HER2 specificity from target-specific experimental supervision and validate predicted variants within that program. Their reported scores should therefore not be treated as measurements of the same transfer capability.

### DiffAb: a structure-conditioned boundary case for sequence–structure co-design

DiffAb is included as a boundary case of the sequence-centric scope because antibody sequence remains part of the designed output while explicit 3D target context is introduced [31]. The method conditions on antigen structure and an antibody framework scaffold and jointly generates CDR residue identities and backbone geometry. Under Axis 2, this sequence–structure output represents substantially greater molecular commitment than scalar scoring or CDR sequence generation alone. The original study trains and evaluates on antibody–antigen structural data and reports computational design, structural, and energy-based metrics across co-design and optimization tasks. Its evidence profile is COMP: generated geometries, docking-related hypotheses, and biophysical energy proxies are computational evidence rather than experimentally resolved STR or independent BIO evidence. The key analytical limitation is therefore proxy alignment. DiffAb directly incorporates geometric context, but stronger conditioning and a thicker output do not themselves demonstrate that generated candidates bind experimentally or reproduce a proposed complex structure.

### AB-Gen: implicit target optimization through multi-objective rewards

AB-Gen illustrates generator–optimization without an explicit antigen input [35]. A repertoire-trained generative prior proposes CDR-H3 sequences, while reinforcement learning shifts generation using weighted reward functions that include a HER2-specificity predictor and developability-related computational proxies. Axis 1 is therefore best interpreted as implicit target conditioning through an external reward. Axis 2 remains restricted to CDR-H3, keeping the framework and other CDRs outside the generated object. The HER2-targeted case study filters generated libraries using predicted properties and examines selected sequence patterns in a Herceptin–HER2 structural context through molecular simulation. The evidence profile is COMP because target specificity and developability are represented by predictors or other *in silico* proxies rather than independent assays on generated candidates. The major limitation is reward dependence: optimization can improve the objective supplied to the agent, but the resulting claim is only as transferable as the reward–assay relationship, and CDR-H3-only generation leaves compatibility with the remaining antibody context to downstream validation.

### BetterBodies: oracle-guided CDR-H3 optimization

BetterBodies provides a related but analytically distinct example of implicit target conditioning [37]. The method generates CDR-H3 sequences using reinforcement-learning-guided latent diffusion, while target-specific optimization is supplied by affinity rewards from the Absolut! simulator rather than by antigen sequence or structure as direct model input. Its output is therefore a local sequence object, whereas the optimization signal is a fixed-target computational oracle. The study reports improved oracle-derived free-energy distributions for SARS-CoV spike RBD and a second antigen case and examines the trade-off between stronger reward guidance and sequence diversity. The evidence profile is COMP. No prospective binding assay independently tests the generated candidates in the original study. Under the three-axis framework, BetterBodies therefore highlights a central distinction between target awareness and target exposure: a generator can be strongly target optimized without directly encoding the target. Its major limitation is proxy dependence, together with the risk that aggressive optimization of the oracle can narrow diversity or exploit model-specific reward structure.

#### Summary (3.7–3.9)

DiffAb, AB-Gen, and BetterBodies all propose new molecular content under target-related objectives, but target context enters through explicit antigen geometry in DiffAb and through predictor- or simulator-derived rewards in AB-Gen and BetterBodies. Their original validation profiles remain computational. The comparison separates conditioning richness from independent evidence: stronger geometric context or target-specific optimization does not itself establish experimental binding or candidate utility.

## Cross-model comparison

This section applies the three-axis framework to recurring comparability problems in the reviewed literature. We distinguish observations demonstrated in individual studies or benchmark analyses from synthesis-derived recommendations of this review. The latter are presented as audit guidance for interpreting cross-study claims rather than as formal community standards.

### What Axis 1 (conditioning interface) controls in practice: claim scope, generalization unit, and assay alignment

Differences in target conditioning become operationally important when a study claims target-specific performance or transfer. Antibody-only foundation priors can support representation reuse or library proposal, but antigen-specific claims depend on the downstream task in which target information is later introduced. Methods conditioned only through target-specific labels, rewards, or external oracles may be useful within a fixed program, but their external validity depends on transfer of that supervision or proxy beyond the context in which it was constructed.

Several recent antibody studies and benchmarks use biologically grouped partitions to test forms of generalization that are not visible under random sequence- or pair-level splits. Taken together, these designs motivate a claim-aligned split audit. Random pair partitions can remain informative for within-regime discrimination, whereas transfer across antibody lineages, related CDR motifs, antigen groups, or epitopes can be examined using the corresponding family- or cluster-aware holdouts. dyMEAN uses CDR-H3 clustering in its partitioning and removes training antibodies from clusters represented in the test benchmark; AbAgIPA separates highly similar antigen subsets; and AsEP evaluates unseen epitope groups [26, 28, 33]. Temporal partitions provide a complementary regime when the claim concerns prospective or future-target generalization.

Interaction-prediction studies also show that split design and synthetic negative construction jointly define the benchmark regime [19, 20, 22, 26]. As a framework-derived audit recommendation, we suggest identifying the biological relationship relevant to the intended claim before constructing synthetic negatives. Groups can then be assigned to train, validation, and test partitions, after which negatives are constructed within partitions or checked explicitly for bridges across the held-out relationship.

Conditioning also affects assay alignment. If a method is conditioned on an epitope peptide, peptide binding is a direct test of the represented target context, whereas full-protein binding or neutralization additionally depends on epitope accessibility and conformation. For implicit target conditioning, interpretation likewise depends on how closely the supervision or optimization signal corresponds to the downstream assay.

### What Axis 2 (output granularity) controls in practice: molecular commitment and evaluation breadth

As molecular output becomes more complete, the uncertainty delegated to a fixed scaffold or downstream pipeline changes. Embeddings and scalar scores leave the antibody unchanged and are evaluated primarily as representation or ranking tools. CDR-H3-only generators commit to a local sequence while leaving compatibility with other CDRs, framework context, and VH–VL geometry to downstream screening. Multi-CDR, joint sequence–structure, and paired VH/VL outputs commit to progressively more of the candidate and therefore expose a broader set of folding, pairing, and candidate-level properties to evaluation.

For generative methods, a central distinction is between generation quality and candidate usability. Library-level quality can be characterized through validity, novelty relative to a defined reference set, diversity, redundancy, and satisfaction of design constraints. Functional utility concerns binding or affinity and, when claimed, discrimination among relevant antigens, epitopes, or competitors. Candidate viability concerns properties such as expression or production feasibility, stability, aggregation or solubility, and other task-relevant developability liabilities. Predicted properties and independent experimental measurements should be reported separately.

The breadth of evaluation can therefore be matched to both output granularity and the stated claim. A CDR-only library proposal may be adequately characterized by a transparent library-quality panel plus functional evidence aligned with any target-specific claim. A near-full-length or paired Variable Heavy Chain/Variable Light Chain (VH/VL) generator presented as a candidate-discovery or developability-oriented system carries a broader molecular commitment; binding, specificity, and relevant candidate-viability dimensions become correspondingly important, or should be identified explicitly as unevaluated. Reporting the number of generated, selected, produced, tested, and successful candidates is also informative because selected successes cannot be interpreted without the relevant attrition denominator. Novelty and repertoire likeness remain useful generation metrics, but they should not be used as surrogates for candidate usability.

### What Axis 3 (validation evidence profile) controls in practice: claim alignment and evidence complementarity

Similar numerical improvements can support different conclusions depending on whether they arise from internal computational evaluation or independent experimental evidence. Held-out Area Under the Receiver Operating Characteristic Curve (AUROC), regression performance, or computational energy improvement can support ranking or prediction under a benchmark’s assumptions, but they do not by themselves establish prospective assay performance. Conversely, experimentally derived labels remain supervision rather than independent validation when the same signal is used to fit or directly optimize the model.

Independent evidence domains are claim dependent. Quantitative binding measurements align with affinity or kinetic claims; antigen, epitope, or competitor panels support specificity; experimentally resolved complexes test binding-mode or structural hypotheses; and developability assays address the properties actually measured. A candidate may therefore have experimentally confirmed affinity without demonstrated specificity or a validated binding pose without characterized manufacturability. The evidence profile keeps these conclusions separate, so that each claim can be interpreted in relation to the type of support actually provided.

Computational proxies remain valuable for triage and optimization, but the source of an apparent improvement should be explicit. When a score is interpreted operationally as a probability, used with an absolute threshold or used to allocate candidates by confidence, calibration or uncertainty reporting becomes relevant; systematic antibody-specific evidence on calibration remains limited, so we treat this as a decision-score reporting consideration. More generally, optimization signals, internal evaluation, and independent validation should be reported separately.

### Framework-guided reinterpretation of benchmark conclusions

The practical test of the coordinate system is whether a headline comparison changes meaning after conditioning, output, and validation assumptions are made explicit. The framework audits whether apparently comparable results address the same generalization problem, molecular object, and claim. Four recurring scenarios illustrate this role.

#### Case 1: High interaction-prediction performance versus target-level generalization

Interaction predictors are often compared directly by AUROC or accuracy. Sardar *et al.* construct nonbinding nanobody–antigen examples largely by exchanging antigens between known pairs and then use repeated random train–test partitioning; NABP-BERT evaluates a processed dataset from the same broad lineage through pair-level partitioning [20, 22]. These designs can measure discrimination within the resulting data regime, but a high score does not by itself demonstrate transfer to biologically distinct targets. AbAgIPA instead groups highly similar antigens and separates antigen subsets between training and test data, while newer benchmarks distinguish unseen epitope, target-group, or temporal regimes [26, 28, 48]. The framework-guided conclusion is not that a lower score under a grouped split indicates a weaker model, since the scores may estimate different capabilities. The relevant benchmarking question changes from ‘Which AUROC is higher?’ to ‘Which generalization problem does each AUROC estimate?’

#### Case 2: Sequence recovery versus functional antibody design

Amino acid recovery and structural Root-Mean-Square Deviation (RMSD) are intuitive reconstruction metrics, but Axis 2 asks whether the measured object matches the functional claim. dyMEAN shows that a position-specific unigram pattern from the training distribution can already achieve substantial CDR-H3 recovery and that removing several highly predictable terminal positions materially lowers the model’s reported recovery [33]. AbBiBench raises a related issue by treating the antibody–antigen complex, rather than antibody-only similarity, as the functional unit for affinity-oriented evaluation [29]. The framework therefore narrows ‘higher recovery indicates a better antibody designer’ to a more specific statement: higher recovery indicates closer reconstruction under a particular distribution and metric. Functional design superiority requires an evaluation object aligned with antibody–antigen interaction or the downstream candidate claim.

#### Case 3: Computational objective improvement versus independently demonstrated candidate performance

AB-Gen uses predictor-derived target and property objectives, BetterBodies uses affinity-related rewards from the Absolut! simulator, and DiffAb reports computational structural and energy-related design criteria [31, 35, 37]. These studies can demonstrate movement toward the supplied predictor, oracle, or computational objective, but such gains do not by themselves establish measured binding, specificity, or agreement with an experimentally resolved complex. By contrast, PALM-H3 supplements generation with *in vitro* binding–related assays, MAGE reports experimental antigen-specific binding for generated paired-chain antibodies, and recent structure-conditioned *de novo* design studies combine experimental binding, epitope-selectivity analyses, or experimentally resolved complexes for selected candidates [36, 39–42]. The framework changes the generic claim ‘improved affinity’ into source-specific statements—improved predicted affinity, independently measured binding, or experimentally demonstrated specificity—which should not be treated as interchangeable.

#### Case 4: sequence novelty versus candidate usability

Nonoverlap with a training set, diversity, and repertoire likeness are useful for detecting memorization, redundancy, or mode collapse, but they answer a library-generation question [9, 15, 38, 43]. Exact train-set novelty does not establish target binding, and predicted solubility, aggregation, or humanness remain computational triage signals rather than measured candidate properties. This distinction becomes more consequential as outputs approach full or paired antibody candidates. For a CDR-only library, validity, novelty, diversity, and constraint satisfaction may characterize generation quality; for a paired VH/VL system presented as a candidate-discovery platform, sequence novelty alone provides limited evidence of usability unless relevant functional and candidate-level properties are tested or identified as unmeasured. Attrition denominators further determine whether a small set of successful designs represents a high hit rate or extensive downstream filtering.

Together, these cases demonstrate the analytical rather than purely taxonomic role of the framework. Axis 1 can narrow a transfer claim when the split or negative regime does not match the implied generalization unit; Axis 2 can reveal that a metric measures reconstruction rather than the claimed functional object; and Axis 3 can separate optimization of a computational proxy from independent experimental support. The combined use of Axes 2 and 3 further separates generation novelty from candidate usability. It does not determine universal model superiority or replace task-specific benchmarks but could expose assumptions that change the interpretation of headline results—what the model sees, what it commits to, and what the study demonstrates.

The operational reporting items used to audit these assumptions, together with concrete reporting examples and common ambiguities, are summarized in Table 2.

**Table 2:** Operational reporting and audit checklist for evidence-aware comparison of antibody machine-learning studies. The checklist specifies minimum information, reporting examples, and common ambiguities across conditioning, output, benchmark construction, generative evaluation, validation, attrition, and code/model/data availability.

Reporting item	Minimum information to report	Adequate reporting example	Ambiguous or insufficient reporting	Why it matters for comparison
Scope and inclusion status	State whether the study is treated as a core sequence-centric antibody ML method, a benchmark or resource, or a boundary case.	Included as a structure-conditioned boundary case because antibody sequence is generated or optimized as the candidate object.	Listing a method without clarifying whether it belongs to the core corpus, a benchmark set, or a boundary case.	Prevents selected examples from being mistaken for an exhaustive model inventory and clarifies how each study is used in comparison.
Input objects and modalities	Report the antibody input object, target input object, modality of each input, and whether chains, epitopes, antigens, or structures are represented directly.	Input: paired VH/VL amino-acid sequences and an antigen epitope peptide; no 3D structure is used.	Describing a model as target-aware without specifying what target information is provided to the model.	Determines whether models receive comparable biological information.
Target-conditioning route	Specify whether target context is absent, provided as direct input, introduced implicitly through labels or rewards, supplied by an external oracle, or used only during filtering.	The antigen sequence is not a model input; target specificity enters through a HER2-specific predictor used during optimization.	Calling a method antigen-specific when target information is only present in the training labels or reward function.	Separates direct target exposure from target-specific optimization and shapes the relevant generalization claim.
Structural-information role	Report whether structure is used as model input, generated output, external guidance, post-generation filter, benchmark label, or computational evaluation only.	Antigen backbone coordinates are model inputs, and CDR sequence plus backbone coordinates are generated outputs.	Using the term structure-aware without specifying whether structure is input, output, supervision, or evaluation.	Structure use affects data dependence, output commitment, and evidence interpretation.
Paired heavy-light-chain handling	State whether the method uses VH only, VL only, VHH/nanobody, separate VH, and VL chains, paired VH/VL chains, or paired-chain generation.	The model generates paired human VH and VL variable regions, and pairing is part of the output.	Reporting antibody generation without specifying whether the light chain is modeled or fixed.	Paired-chain commitment changes candidate realism and the evaluation burden.
Output object and editable regions	Define the exact molecular or decision object scored, ranked, or generated, including fixed scaffold regions and editable CDR or framework regions.	Generated variable-length CDR-H3 sequences while the scaffold and other CDRs were fixed.	Reporting antibody design without defining whether only CDR-H3, multiple CDRs, variable regions, or paired chains are produced.	Ensures that metrics are interpreted in relation to the object the model actually commits to.
Training and evaluation data source	Report data source, snapshot, or access date when relevant, curation criteria, redundancy filtering, label origin, and train/evaluation separation.	Training data were drawn from OAS; sequences with nonstandard amino acids were removed, duplicates were filtered, and held-out test data were separated before model fitting.	Using public antibody data without stating database version, filtering, or label source.	Dataset construction can dominate apparent performance and generalization.
Data scale and denominators	Report numbers of antibodies, sequences, antigens, epitopes, complexes, assays, positives, negatives, and candidates at each relevant stage.	The benchmark contained 2922 antibody–antigen complexes; 43–101 designs were experimentally tested per antigen.	Reporting only a headline training size or a number of successful candidates.	Scale and denominator determine how performance, success rate, and attrition should be interpreted.
Split unit and biological grouping	Report split unit, grouping variable, clustering rule, or threshold if used, partition counts, and whether related biological groups are blocked across partitions.	Antigens were clustered by sequence similarity, and all members of a cluster were assigned to the same train, validation, or test partition.	Reporting a random 80/10/10 split without saying whether the split is by pair, antibody, antigen, epitope, or cluster.	The split unit determines whether a metric estimates interpolation, lineage transfer, unseen-target transfer, or another generalization regime.
Temporal split	If prospective or time-forward generalization is claimed, report data cutoff date, source update policy, possible pretraining overlap, and temporal assignment rule.	Training used complexes deposited before 1 January 2023, whereas the test set used later-deposited complexes that passed the same curation criteria.	Claiming a latest or prospective test set without specifying cutoff date or possible leakage through pretraining data.	Temporal splits test a different claim from random or cluster holdouts.
Synthetic negative construction	Report negative source, experimental versus synthetic status, sampling rule, positive-to-negative ratio, hard-negative criteria, and relation to the split unit.	For each positive pair, two negatives were sampled from antigens outside the held-out antigen cluster; negatives were constructed after train/test partitioning.	Stating that negative samples were randomly generated without describing the sampling rule.	Negative sampling can introduce shortcuts or reconnect held-out biological groups across splits.
Benchmark type	Distinguish internal held-out evaluation, cross-validation, external independent datasets, temporal tests, community benchmarks, simulator benchmarks, and experimental validation sets.	Evaluation used an antigen-cluster-held-out test set, and AUROC was computed over synthetic nonbinding pairs generated within the test partition.	Reporting AUROC without specifying benchmark regime.	The same metric can support different conclusions under different benchmark designs.
Evaluation metric definition	Define metric, evaluation object, positive/negative class, thresholding rule, aggregation level, sequence region, and any contact or structure definition.	Amino-acid recovery was computed over CDR-H3 residues; contact recovery used residues within 6.6 Å of the antigen in the reference complex.	Reporting higher recovery without defining the sequence region or contact criterion.	Prevents metric names from hiding different measured objects.
Probability calibration and uncertainty	When scores are interpreted as probabilities or used with absolute thresholds, report calibration method, validation set, calibration metrics, and uncertainty estimates when available.	Temperature scaling was fitted on validation data; expected calibration error and reliability curves were reported on the held-out test set.	Using predicted probability as an absolute binding probability without calibration or uncertainty assessment.	Operational candidate allocation depends on score reliability, as well as ranking performance.
Generative novelty	Report novelty definition, reference set, sequence region, identity, or distance threshold, and whether exact matches, or near matches are excluded.	Novelty was defined as the fraction of generated CDR-H3 sequences with no exact match in the training set or OAS reference set.	Reporting novelty without a reference set or threshold.	Novelty relative to training data is not equivalent to functional novelty or candidate usability.
Generative diversity and redundancy	Report diversity metric, duplicate rate, clustering threshold, sampling temperature, and whether diversity is computed before or after filtering.	Diversity was measured by mean pairwise Levenshtein distance over unique generated CDR-H3 sequences after duplicate removal.	Describing a library as diverse without a metric or duplicate-handling rule.	Diversity can be inflated or reduced by sampling settings and filtering choices.
Functional utility evaluation	For target-aware claims, report whether binding, affinity, enrichment, or another assay was measured directly or predicted computationally; define target panel and success threshold.	The top 20 generated candidates were tested by BLI against the target and two related off-target antigens.	Reporting that generated candidates were predicted to bind without an assay or off-target panel.	Binding, affinity and specificity are different candidate claims and require different evidence.
Candidate viability and developability	Report measured or predicted expression, stability, aggregation, solubility, viscosity, polyspecificity, humanness, immunogenicity risk, or other claimed candidate-level properties.	Expression yield and SEC aggregation were measured for all expressed candidates and reported together with binding results.	Claiming improved developability from an unspecified computational score.	Candidate-like outputs require practical progression evidence beyond sequence validity or novelty.
Candidate selection rule for validation	Report whether validated candidates were top-ranked, randomly sampled, diversity-selected, manually selected, or filtered by additional criteria.	Thirty candidates were randomly selected from the high-scoring pool after applying the prespecified developability filter.	Stating that representative candidates were validated without explaining how they were chosen.	Selection rule determines whether validation estimates ranking quality, library quality, or only selected-case performance.
Experimental attrition chain	Report generated, filtered, selected, synthesized, or constructed, expressed, purified, tested, and successful candidate counts where applicable.	100 000 candidates were generated; 2000 passed filters; 50 were selected; 45 were synthesized; 39 expressed; 32 were tested; 6 met the binding threshold.	Reporting only that six binders were identified.	Successful examples cannot be interpreted without the denominator and failure stages.
Experimental assay reporting	Report assay type, target format, concentration, or titration range, replicate structure, success threshold, measured parameters, and statistical uncertainty where applicable.	BLI measured KD, kon, and koff for expressed candidates using full-length antigen across a specified titration range with biological replicates.	Saying that binding was confirmed experimentally without assay details.	Assay details determine whether evidence supports quantitative binding, enrichment, specificity, or developability claims.
Specificity panel	If specificity is claimed, report target, epitope, or competitor panel, relatedness of off-targets, negative controls, and panel-selection rationale.	Candidates were tested against the target antigen, a homologous antigen, an epitope-overlap competitor, and an unrelated control antigen.	Claiming specificity after showing binding to one target only.	Single-target binding does not establish antigen, epitope, or competitor specificity.
Structural validation	Report whether structural evidence is predicted, docked, simulated, or experimentally resolved; if resolved, report method, resolution, or quality and comparison to design model.	A cryo-EM structure of the candidate–antigen complex confirmed the designed binding mode for the selected candidate.	Calling a docking pose structural validation.	Predicted complexes are structural hypotheses, whereas experimentally resolved complexes provide independent structural evidence.
Resource availability	Report availability of raw data, processed data, split files, negative-pair lists, or generation scripts, source code, pretrained weights, inference configuration, and license restrictions.	Code, pretrained weights, processed train/validation/test files, and generated negative-pair lists are available in a versioned repository.	Reporting code availability while omitting processed splits or negative-pair construction scripts.	Raw data alone may not reproduce benchmark claims or model comparisons.
Claim wording	State whether results support representation reuse, within-regime ranking, target-specific optimization, unseen-target transfer, candidate generation, or experimentally validated discovery.	The model supports within-regime antibody–antigen pair discrimination under constructed negatives; unseen-antigen transfer was not tested.	Claiming binder discovery from computational score improvement alone.	Keeps the claim aligned with the evaluated data regime, output object, and evidence profile.

## Discussion

The sequence-centric scope of this review intersects an increasingly multimodal field. Recent antibody models combine sequence representations with 3D geometry, molecular interfaces, structural constraints, or learned protein representations, and these signals can enter a workflow through joint sequence–structure modeling, feature-level fusion, external structural guidance, or post-generation filtering [31–34, 39–42, 46, 51]. The three audit questions remain useful across these systems, but Axis 1 does not fully capture how multiple modalities are integrated. Methods with similar target context may therefore have different data dependencies and failure modes. Modality-integration strategy is a plausible additional descriptor for future benchmarking, although current approaches and reporting conventions remain too heterogeneous to justify a universal fourth axis.

The independent evidence domains can also form a ladder-like or funnel-like progression in practical antibody discovery. Computational triage and scalable selection are typically applied to large candidate pools, whereas quantitative binding, specificity characterization, and deeper structural or developability assays are generally performed on smaller sets as experimental cost and decision commitment increase. This operational progression helps explain how evidence accumulates during candidate advancement, but it should not be interpreted as a universal hierarchy of evidential strength. Affinity, specificity, structural validation, and developability support different claims and may be acquired in parallel, iteratively, or in different orders depending on the intended application and development stage.

A related challenge is that antibody generation and optimization are intrinsically multi-objective. Novelty, binding, specificity, and candidate-level biophysical properties can create different selection pressures, and success on a single computational reward does not imply favorable behavior across the remaining dimensions. Reporting attrition from generated libraries to produced, tested, and successful candidates can therefore be as informative as reporting top designs, particularly when a method is presented as a practical discovery system.

The framework should nevertheless be interpreted as a review-derived audit scaffold rather than an empirically validated community standard or exhaustive ontology. Architecture, data scale, computational cost, modality integration, industrial constraints, and regulatory context may provide important additional views. The present three axes were retained because they expose recurrent assumptions about problem context, molecular commitment, and supporting evidence across heterogeneous method classes. Their value lies in making those assumptions visible and in showing when a headline comparison must be narrowed, not in assigning every future model a unique coordinate or guaranteeing improved reproducibility. As antibody machine learning evolves, additional descriptors should complement this core audit rather than be forced into a fixed taxonomy.

## Conclusion

Sequence-centric machine learning now spans repertoire-scale foundation priors, target-aware scorer–rankers, and generator–optimizers that propose increasingly complete antibody designs. Our structured mapping shows that the principal barrier to cross-study interpretation is not simply architectural diversity. Similar task labels and headline metrics can correspond to different target-conditioning regimes, molecular outputs, split units, negative constructions, and validation evidence. The three-axis framework is therefore used here as an analytical audit scaffold: conditioning interface identifies the biological context available to a method, output granularity records its molecular commitment, and the validation evidence profile separates internal computational evaluation from complementary domains of independent experimental support.

Applying this scaffold changes the interpretation of several recurring comparisons. Pair-level discrimination should not be equated with transfer to held-out target groups; sequence recovery does not by itself establish functional design superiority; optimization of an affinity or energy proxy is not equivalent to measured binding or specificity; and sequence novelty is distinct from candidate usability. The practical implication is not a universal benchmark recipe, but a need to report the data regime, evaluation object, and source of supporting evidence with enough precision for like-with-like comparison. As multimodal and paired-chain design systems mature, standardized method extraction, transparent attrition reporting, and claim-aligned validation will become increasingly important. A tighter connection between modeling assumptions and experimental interpretation should make antibody machine-learning studies easier to audit, compare, and translate into discovery workflows.

**Key Points**

- Sequence-centric antibody machine-learning studies can report similar metrics while testing different target-conditioning, generalization, and validation regimes.
- The reviewed methods are organized into foundation priors, scorer–rankers and generator–optimizers, and compared through conditioning interface, output granularity, and validation evidence profile.
- The framework is an analytical audit scaffold: it asks what the model sees, what molecular object it commits to and what the study demonstrates.
- Recurrent comparability problems include biologically permissive splits, synthetic-negative shortcuts, output–metric mismatch, proxy–assay mismatch, and conflation of sequence novelty with candidate usability.
- Standardized method extraction and operational reporting of splits, negatives, evaluation metrics, validation domains, attrition, and resource availability support more transparent cross-study interpretation.

## Data Availability

No new data were generated or analyzed in support of this review.
